# Food-Based Dietary Guidelines for the Arab Gulf Countries

**DOI:** 10.1155/2012/905303

**Published:** 2012-01-11

**Authors:** Abdulrahman O. Musaiger, Hamed R. Takruri, Abdelmonem S. Hassan, Hamza Abu-Tarboush

**Affiliations:** ^1^Nutrition and Health Studies Unit, Deanship of Scientific Research, University of Bahrain, Bahrain; ^2^Arab Center for Nutrition, P.O. Box 26923, Manama, Bahrain; ^3^Department of Nutrition and Food Technology, Faculty of Agriculture, The University of Jordan, Amman 11942, Jordan; ^4^Department of Health Science, Human Nutrition Program, Qatar University, Qatar; ^5^Department of Food Science and Nutrition, College of Food and Agriculture, King Saud University, Riyadh 11451, Saudi Arabia

## Abstract

The concept of food-based dietary guidelines (FBDG) has been promoted by several international organizations. However, there are no FBDG for the countries in the Arab region. As the Arab Gulf countries share similar a socioeconomic and nutrition situation, an attempt was made to develop FBDG for these countries. This paper summarizes the steps taken to develope such guidelines by the Arab Center for Nutrition. The FBDG were developed through 6 steps: (1) determination of the purpose and goals for establishing FBDG, (2) characteristics of FBDG, (3) determination of the food consumption patterns, (4) review the current nutrition situation, (5) determination of the lifestyle patterns that are associated with diet-related diseases and (6) formulating the FBDG. The FBDG consist of 14 simple and practical pieces of advice taking into consideration the sociocultural status and nutritional problems in the Arab Gulf countries. The FBDG can be a useful tool in educating the public in healthy eating and prevention of diet-related chronic diseases.

## 1. Introduction

The Arab Gulf countries, namely, Bahrain, Kuwait, Oman, Qatar, Saudi Arabia, and United Arab Emirates have undergone a rapid change in their socio-economic situation, food consumption patterns, and lifestyle and health status during the past four decades. This was mainly caused by the sharp increase in income due to accumulated oil revenues. Communicable diseases have almost diminished and diet-related chronic diseases have become the main health problems. However, undernutrition and micronutrient deficiencies still exist, especially among vulnerable groups [1].

The concept of food-based dietary guidelines (FBDG) has been promoted by the Food and Agriculture Organization (FAO) and the World Health Organization (WHO) of the United Nations since the 1992 International Conference on Nutrition in Rome, Italy. One of the recommendations of this conference was that individual countries should develop simple dietary guidelines that are based on their specific public health concerns and relevant to people of different ages, lifestyles, and cultures [2].

The action needed to promote healthy nutrition and a healthy lifestyle has been emphasized at several meetings in Arab Gulf counties [2, 3]. In 1997, the first Workshop on Diet, Nutrition and a Healthy Lifestyle in the Arab Gulf countries was held in Manama, Bahrain. Two main recommendations were proposed: establishing food-based dietary guidelines (FBDG) for the Arab Gulf countries, and promoting a healthy lifestyle in these countries [3]. 

A simplified Arabic version of the FBDG for the Arab Gulf countries was published in a booklet to be used by the public. It was distributed to many health authorities in the region, and it is also available in the website of the Arab Center of Nutrition for more dissemination. Several comments regarding rephrasing and clarifying some statements in this version were received. Therefore an attempt was done to release the second version, including all the comments received from the public, nutritionists, and professionals. 

The main objectives of this paper, therefore, are to summarize the steps taken to develope the food-based dietary guidelines for the Arab Gulf countries, and to explore the scientific bases of these guidelines.

## 2. Methods

**Developing Food-Based Dietary Guidelines (FBDG) for the Arab Gulf Countries**

**Step 1 (Determine the Purpose and Goals for Establishing FBDG)**

The Arab Gulf countries are facing great challenges to prevent and control several nutritional problems and diet-related chronic diseases. Two types of nutritional and health problems occur: those associated with change in lifestyle, such as obesity, cardiovascular disease, diabetes, hypertension, cancer, dental caries, and osteoporosis and those associated with nutrient deficiencies, such as iron deficiency anemia, undernutrition among preschool children, and deficiencies of vitamin D and calcium. In addition, food-borne diseases are a problem of concern in these countries [1]. Therefore, the need for simple dietary guidelines to address the burden of these diseases is urgent, especially as some of these diseases contribute to more than 50% of total mortality in this region [4].

There are no specific food-based dietary guidelines used by Arab countries in general. Nutritionists, dietitians and other health workers in the Arabian Gulf region have been relying on FBDG developed for other countries, such as USA, Canada, and UK, to convey nutrition messages to the public.

In Oman, an attempt was made to establish guidelines to healthy eating. However, these guidelines lack some issues related to the prevention and control of diet-related chronic disease such as reduced intake of salt and added sugar in foods, weight maintenance, and avoiding alcoholic drinks and smoking. Encouraging drinking of water and other liquids were given very little attention; it was mentioned in conjunction with physical activity, without any further advices. In general, these guidelines were prepared for the people who are responsible to educate the public, but not directed to the public [5]. In general our work is different than Omani guidelines in two main issues: (1) the Omani guidelines were prepared for the health workers and not for the public, and (2) the Omani guidelines were focused in portion size of the food groups while the current guidelines focused on food-based advices.

An attempt, therefore, has been made to develop FBDG specifically for Arab Gulf countries to provide simple and practical dietary guidelines to help prevent and control the main nutrition-related disorders and that take into account the socio-cultural, lifestyle, and dietary habits of the people in these countries.

**Step 2 (Characteristics of FBDG)**

To successfully promote healthy eating habits, a number of specific characteristics of FBDG were identified based on current knowledge and available information on food, nutrition, and health status in the countries of the region. These characteristics can be summarized as follows [2, 6].

1. The guidelines should address the prevention and control of the main nutritional problems and diet-related diseases in the Arab Gulf countries.
2. They should be based on affordable and available foods, which are commonly consumed by the public, including traditional foods.
3. They should consider the local dietary habits and food consumption patterns in the Arab Gulf countries.
4. They should take into account food safety situation.
5. They should consider the cultural and religious background of the population in Arab Gulf countries.
6. They should consist of short, clear, and simple messages.
7. They should promote a healthy lifestyle, especially the promotion of physical activity.
8. They should be based on current scientific and health information.

**Step 3 (Determine the Food Consumption Patterns)**

A systematic literature review of the studies published in English between 1990 and 2010 was carried out, using PubMed and Google Scholar search to find out the food consumption patterns and nutrition situation in the Arab Gulf Countries. The key words used were food consumption, nutrition, diet, anaemia, iodine deficiency disorders, cardiovascular disease, diabetes, vitamin D deficiency and undernutrition for each country. Over 800 papers and reports were identified primarily. About 120 papers that possibly addressed food and nutrition in the Arab Gulf states were then identified to be included in the main review. However, for the purpose of this report, 40 papers were selected from these 120 papers to give general background on the food and nutrition status in these states.

There has been a drastic change in food consumption patterns in the Arab Gulf countries. This change includes both quantitative and qualitative change in diet. The structure of diet has shifted towards a high-energy-density diet with more fat and added sugar in foods, more saturated fat (mostly from animal origin) and lower intake of complex carbohydrates, dietary fiber, fruit, and vegetables [1]. For example, the total per capita energy intake exceeds 3000 kcal in all Arab Gulf countries, and the fat represents 25–35% of total energy. However, animal fat represents 40–52% of total per capita fat intake in the region [7].

The intake of animal source foods is growing steady and it replaced many typical diets in the region [8]. In general, poultry and eggs were more consumed compare to red meat, and fish less than that of poultry and eggs. Data from FAO Food Balance Sheets indicated that the daily per capita availability of poultry meat during 2003–2005 ranged from 106–163 grams in the Arab Gulf countries, compared to 2–73 grams in other countries in the Middle East. There was a slight increase in fish intake during 1990–2005; however, the average daily per capita availability of fish was still low compared to red meat and poultry (ranged from 24 to 52 grams) [7]. Although the total production of milk and dairy products is increasing in the Gulf Region during the past decade, the per capita intake of these foods is still below the daily requirements. The daily per capita availability of milk and dairy products has decreased by 20% and 33% in Kuwait and UAE, respectively, and increased by 15% in Saudi Arabia, during 1990–2005 [9].

The intake of fiber-rich foods is particularly important, as the relationship between dietary fiber and prevention of some chronic diseases is well established [10]. Since fiber is only available in foods of plant origin, the intake of fruit, vegetables, and complex carbohydrates is a good indicator for fiber intake. The consumption of fruit and vegetables by people in this region is below the recommended allowances. The WHO/EMRO reported that more than 85% of adults in the Arab Gulf countries consumed fruit and vegetables below 5 servings per day [11]. Additionally, the consumption of whole grain in this region is decreasing markedly, with more dependence on refined cereals [12]. In Saudi Arabia, for example, it was found that the main contribution to fiber intake came from vegetable-based foods (31%), followed by cereal and their products (26%), and fruit and their products [13].

Food rich in salt is highly consumed in the region. Data available from food composition of dishes commonly consumed in the Arab Gulf countries showed a high content of sodium in these dishes [14]. The high use of table salt, spices, and pickles, in addition to the salinity of water are contributing factors for the high intake of sodium in these countries [15]. Among children and adolescents, the high consumption of fast foods and French fries is playing a great role in the increasing intake of sodium among these age groups [16].

High intake of foods rich in added sugar, particularly among children and adolescents, has been reported by many studies in the region [17–19]. Potential health problems associated with overconsumption of food rich in free sugar such as overweight, dental caries, and potential enamel erosion are well documented [20]. In Saudi Arabia, for example, carbonated beverages and canned fruit drinks represented 26% and 25% of daily fluid consumption by adolescents, respectively [21].

**Step 4 (Determine the Food and Nutritional Status)**

Undernutrition, manifested by stunting, wasting, and underweight, is still a problem of concern among preschool children (1–5 years) in the region. The prevalence of stunting in these children ranged from 18% to 22% whereas wasting ranged from 6% to 13%, and underweight ranged from 7% to 23% [8, 22].

Despite the high per capita income in the Arab Gulf countries, anemia especially iron deficiency anemia, remains an important nutrition problem. According to WHO criteria for hemoglobin, the prevalence of anemia was 20%–60% among preschool children, 13–50% among school children and adolescents, and 23%–54% in women of childbearing age [23]. Low intake of dietary iron as well as foods enhancing iron absorption is the most possible factor that contributes to iron deficiency anemia [24].

Vitamin A deficiency is less common compared to other micronutrient deficiencies. It was estimated that the prevalence of vitamin A deficiency among 0–72-month-old children ranged from 16% to 20% [22, 25]. Iodine deficiency disorders are not a problem of concern in most Arab Gulf countries. They are mostly prevalent in the mountain area. The prevalence of goiter in these countries ranged from 2% to 10% [25].

Although these countries have a sunny environment, vitamin D deficiency is one of the main public health problem. In Qatar, for example, it was reported that 69% of children below 16 years had vitamin D deficiency [26]. Studies in Saudi Arabia revealed that 28% to 80% of adults had vitamin D deficiency [27]. Low sunlight exposure, low intake of dietary vitamin D and calcium, and short duration of breastfeeding during the first six months are the main risk factors for occurrence of this problem [26].

The change in dietary habits, lifestyle, and life expectancy in the Arab Gulf countries has led to a remarkable change in disease trends. Diet-related chronic diseases such as cardiovascular disease (CVD), diabetes mellitus, hypertension, obesity, cancer, dental caries, and osteoporosis have become the main health problems [13]. Overweight and obesity have become epidemic among all age groups in the Arab Gulf countries, responsible for increased morbidity [1]. It was estimated that 12%–25% of school children aged 6–11 years were overweight and obese in this region. The proportion increased to 15%–45% among adolescents (11–18 years), and to 30% to 60% among adult men, and to 35% to 75% among adult women [12]. Inactivity, high consumption of high-energy-density foods, multipregnancy, and long duration of watching television or using the internet were reported as contributing factors for high prevalence of obesity [1, 13, 28].

Cardiovascular diseases (CVD) are the main cause of death in the region. Health statistics revealed that 28–30% of total deaths in the Arab Gulf countries were due to CVD [4]. With changes in lifestyle, exposure to risk factors for CVD has become more prominent, such as diets high in saturated fat and low in dietary fiber, hypertension, diabetes, hyperlipidemia, smoking, and inactivity [13].

The prevalence of diabetes among adults in the Arab Gulf countries is higher than that reported in Western Region. The rate of type 2 diabetes among adults ranged from 12% to 23%. Prediabetes and undiagnosed diabetes are particularly important since they can lead to chronic disease complication, if left untreated [29]. In Saudi Arabia, for example, it was found that 28% of diabetes were unaware of their condition [30]. Age is one of the main factors associated with increasing prevalence of type 2 diabetes and impaired glucose tolerance among the Gulf population [31]. Obesity, especially abdominal obesity, is the potent risk factors for the high proportion of type 2 diabetes [32]. In general, diabetes is getting more attention by health authorities in the region, due to health and economic burden on government services [33].

The high risk of diabetes and CVD associated with obesity in the Arab Gulf countries may lead to the metabolic syndrome [34–36]. The prevalence of the metabolic syndrome ranged from 29.6% to 36.2% in men and 36% to 46% in women using International Diabetes Federation definition. Overall, the prevalence of the metabolic syndrome in the Arab Gulf countries is 10–15% higher than in most developed countries [30].

Data on the prevalence of diet-related cancer in the Arab Gulf are mostly hospital based. It was estimated that about 10–12% of total deaths were due to cancer. Breast, bronchial, lung, stomach, colon, and liver cancers are the most common types of cancer which may be related to diet [4].

Osteoporosis is a growing public health problem in the Arab Gulf. Studies in the region showed that a lower bone density was common, especially among women [37–39]. The main risk factors are female sex, age, menopause, smoking and vitamin D and calcium deficiencies [40].

Food-borne diseases are now considered a major public health problem worldwide. The diseases may be toxic or infectious in nature and are caused by the ingestion of contaminated foods. These diseases can be grouped on the basis of causative agents: bacteria, fungi, viruses, parasites, and chemicals. The health consequence due to these diseases can be very severe and may lead to death, especially in individuals with low disease resistance, such as infants, young children, pregnant women, and the elderly. Food-borne diseases in Arab Gulf countries are mainly caused by bacteria, specifically salmonellosis, hepatitis A, shigellosis, and poisoning with staphylococcus are the main constitutes of these diseases [41, 42].

**Step 5 (Determine the Lifestyle Patterns That Are Associated with Diet-Related Diseases)**

In addition to the changes in food consumption patterns, other changes in lifestyle, are also apparent and include smoking, a decrease in physical activity, an increase in alcohol consumption, and drug abuse. The increase in sedentary lifestyles is mainly due to the abundant use of cars, dependency on housemaids for home management, as well as due to spending a long time watching television and using the Internet, particularly by children, teenagers, and young people [13, 43]. The prevalence of adult physically activity for at least 150 min/week (based on the international standard definition) ranged from 30% to 42% for men and 26% to 29% for women. Participation in physical activity in the GCC states was estimated to be considerably lower than those for many developed countries [30].

An increase in smoking amongst both males and females was reported, with the prevalence of cigarette smoking ranging from 20–50% among men, and 5% to 12% among women [44]. The smoking of *shisha* (water pipe) has also increased sharply in the region and become more acceptable in the community [45]. This has increased the rate of smoking among the Arab Gulf population. In addition, passive smoking complicated the problem, as many family members, especially women and children are regularly exposed to a smoking environment at home. It was estimated that 30–40% of women in Bahrain were exposed to a smoking environment in the home [46].

**Step 6 (Formulating and Testing the First Draft of the FBDG)**

After collecting information on nutritional status and lifestyle, the first draft of the FBDG was formulated and reviewed by five nutrition experts in the region. The draft was also pretested on a small number of people in the Arab Gulf countries. The draft was distributed to the nutritionists and dietitians in the region. No major comments were obtained from this limited number of the public. However, several comments and suggestions were provided by the experts. The first draft of the Arabic version was then modified and revised based on the comments received by both the experts and nutritionists.

## 3. Findings and Discussion

**The Food-Based Dietary Guidelines (FBDG) for Arab Gulf Countries**

 

### 3.1. Eat a Variety of Different Foods Every Day

The human body needs more than 40 nutrients to maintain good health and prevent disease. However, there is no single food which contains all these nutrients in their required entirety and in the right quantity to maintain a healthy body. Therefore, increasing the variety of foods consumed is important to ensure an adequacy to intake of these nutrients in our meals, breakfast, lunch, supper, and snacks [47].

As each type of food can be rich in certain nutrients but poor in others, it is important to eat food from all five food groups, as listed in these guidelines, to ensure a varied diet (Table 1). Select one or more type of food and try to combine them in your favorite way in every meal. For example, for lunch, you could select rice from the cereal group to eat with fish (meat group) in addition to vegetables and salad (vegetable group), along with a glass of fruit juice (fruit group). 

### 3.2. Eat an Adequate Amount of Fruit and Vegetables Daily

Fruit and vegetables must comprise a basic part of your daily diet to maintain a healthy body. Their consumption can contribute to the prevention of several diet-related chronic diseases [48–50]. Studies indicate that the majority of the population of Arab Gulf countries does not consume sufficient quantities of fruit and vegetables, particularly children and teenagers, and this deprives them of essential nutrients [14]. It is crucial to vary your daily intake of fruit and vegetables to obtain the maximum nutritional and health benefits of such foods. It is recommended to eat at least three servings of vegetables and two of fruit daily, as shown in (Table 1).

### 3.3. Eat Meat, Fish, Chicken, Legumes, and Nuts Regularly

Meat, fish, and poultry are the main source of dietary balanced protein and are rich in essential nutrients, especially iron, zinc, and other vitamins [51]. Communities in the Arab Gulf region frequently suffer from anemia, mainly iron deficiency anemia [23]. Meat, fish, and chicken are good sources of good absorbable iron. Take one to two servings of fish, skinless poultry, or lean meat daily (as prescribed in the food groups). Increased fish intake is especially important as scientific information showed that the consumption of fish or fish oil containing omega-3 polyunsaturated fatty acids reduces the risk of coronary heart disease [52]. At the same time, red meat consumption should be restricted to not more than 0.5 kg per week, as high consumption of red meat may increase the risk of stomach and colon cancer [53]. Limit your intake of processed meat products that are rich in fat and salt such as sausages and mortadella, and eat low-fat processed meat. Reduce the intake of liver, cerebrums, and kidneys due to their high cholesterol content. Also, reduce the intake of salted fish and fish sauces such as *Mihiyawa (Mishawa)* and *Tareeh* as they contain a great amount of salt [14]. 

Legumes are a rich source of protein, some vitamins, and minerals, and dietary fiber. Legumes include, among others, beans, lentils, cowpeas, kidney beans, lupine, green peas, and soybeans. On the other hand, nuts such as walnuts, hazelnuts, almonds, and pine nuts, as well as seeds such as watermelon seeds, muskmelon, and sunflower are very rich sources of energy for their high fat content, which ranges between 30% to 60% [54]. Nuts and seeds are also important sources of certain vitamins, minerals, phytochemicals, and unsaturated fatty acids that are recommended for their protective role against heart disease [55]. The intake of nuts and seeds is recommended, at least, once to twice a week, using the serving size prescribed in the food groups. Reduce the intake of salty nuts and seeds for their high content of sodium and instead choose unsalted roasted nuts. Nevertheless, it should be noted that overconsumption of nuts can contribute to an increase in calories. 

### 3.4. Make Sure That Your Daily Diet Contains an Adequate Amount of Cereals and Their Products

Arab Gulf communities rely on grain-based foods, mainly rice, as the main food in addition to wheat (which is processed to produce bread, macaroni, pastries, cakes, and other products) [1]. It is important to consume adequate amounts of these foods particularly those made from whole grains—one of the most important sources of fiber and other nutrients. Unfortunately, many people do not consume an adequate amount of food that consists of complex carbohydrates such as whole-wheat products, although it is recommended that at least 55% of calories should come from carbohydrates [8]. 

Try to concentrate on foods based on whole grains especially bread, biscuits, and other bakery products. Cereal-based foods such as cakes, biscuits, and pastries which can have high levels of added fats and sugars are not included in this recommendation and should be regarded as occasional treats only. Active adolescents and youths need more carbohydrates to compensate for daily exerted energy than nonactive adolescents. Foods from grains such as rice, oats and wheat, in addition to carbohydrates supply the body with some vitamins, minerals, and dietary fiber. Additionally, these foods are low in fat except when fat is added during processing or cooking. Whole-grains are different from peeled grains in that the latter lose some of their nutrients and fiber, a fact that reduces their nutritional value. For those who find the taste of whole grains unpleasant or where they are not available, this can be compensated for by eating other types of food like fruit and cooked or fresh vegetables to ensure an adequate intake of dietary fiber. It is recommended that brown or whole grain bread be an essential part of your daily intake, as it is available and affordable. Also, at least half of your daily intake of grains must be whole grains as much as possible [56]. 

Eat fortified grains when possible. This term refers to grains to which vitamins and minerals are added to flour to increase their nutritional value. Hence eat grains fortified with iron, folic acid, calcium, and vitamin D whenever possible. There is considerable interest in the production of grains fortified with vitamins and minerals that compensate for any deficiency resulting from the elimination of these nutrients during processing. The most common nutrients used in fortification are folic acid, iron, and some of the B vitamins. The main fortified grain-based foods available in the region are cornflakes, bread, and some types of biscuit. Read the label on the product to ensure that the foods have been fortified with these nutrients [57].

### 3.5. Consume an Adequate Amount of Milk and Dairy Products Every Day

Milk and dairy products, which contain many essential nutrients, are important for the body and for its development. Dairy products are the best source of calcium, which is vital to strengthen bones and for a healthy nervous system [58]. In general, young female adolescents and elderly people are more susceptible to a “deficiency in calcium” and this is one of the leading causes of osteoporosis—a disease that is currently widely prevalent among Arab Gulf communities [38, 59, 60]. 

 The consumption of milk and dairy products is important for building a healthy body, particularly during childhood and adolescence. Studies of dairy products, particularly yogurt and cheese along with milk, have revealed a positive link between the consumption of dairy products and bone density and the existence of high mineral content [61]. Dairy products should not be avoided on the grounds that they lead to becoming overweight. Children between 2 to 8 years old should consume about 2 cups of low-fat milk or the equivalent each day while nine year olds and above are advised to consume 3 cups of low-fat milk or the equivalent each day. For those who are allergic to milk or who are intolerant to lactose, yogurt or cheese (low fat) are suitable substitute, for milk [51].

### 3.6. Reduce the Intake of Food Rich in Fat

Many foods and traditional dishes consumed in the Arab Gulf region are known for their considerable high fat content (5%–20%) [14], and this does not mean to exclude these dishes, but to reduce the amount consumed. Fat provides energy, essential fatty acids necessary for development and fat helps in the absorption of certain vitamins. Nevertheless, moderate intakes of fat should always be maintained. Dietary fats can be classified into two types, based on their relation to heart disease and their effect on raising cholesterol levels in the blood. Animal fat, which may raise blood cholesterol, is mainly found in meat, chicken skin, cheese, whole-milk butter, and liver. Most of vegetable fats do not increase blood cholesterol [62]. However, remember that fats contain plenty of energy, so excessive intake of food rich in fat may play an important factor for prevalence of obesity in this region [1]. Try to get energy requirements from foods of plant origins such as grains, legumes, seeds, and nuts. If you eat foods which contain a high amount of animal fat at one meal, try to eat foods low in this type of fat in the second meal. Also try to replace meat with fish whenever possible and affordable.

### 3.7. Reduce the Intake of Food and Drink High in Sugar

Sugar is considered a carbohydrate and a source of energy. In general, all carbohydrates (except dietary fiber) are converted to sugars. Natural sugars are found in many foods such as milk, fruit, certain vegetables, bread, grains, and potatoes. Frequent intake of foods rich in added sugar, especially sweets, chocolates, and sugary drinks (such as soft drinks and canned juices) can contribute to tooth decay, unless the teeth are cleaned immediately after eating such foods. Evidence from many research studies suggests that the proportion of tooth decay increases with an increase in the intake of sugar per day [8, 63]. In order to combat teeth decay, you must reduce the intake of food rich in sugar, regularly clean your teeth, and use toothpaste containing fluoride. Overconsumption of food rich in sugar leads to an increase in energy intake, which contributes to an increase in your weight.

### 3.8. Reduce the Use of Salt and Intake of Salty Foods

Dietary salt is an inorganic compound consisting of sodium and chloride ions. It is found naturally in many foods and it is also added to many foods for flavoring and preservation. It has been found that Arab Gulf people consume more sodium than they need [13]. Many Gulf traditional dishes, canned, and fast foods contain high amounts of salt. Studies show that an overintake of sodium is associated with high blood pressure, strokes, and contributes to heart attacks, heart failure, and kidney failure [64, 65]. It is recommended not to exceed 5 g sodium per day [8]. To achieve low intake of salt, people should consume fresh foods, foods normally processed without salt, and add low salt or avoid addition of salt to food. In case of using salt, use iodized salt. Among the substitutes for salt are acidic ingredients such as vinegar, lemon, line, spices, and herbs [51]. 

### 3.9. Drink Adequate Amounts of Water and Other Liquids Daily

Water is an essential nutrient for life. It accounts for more than 60% of our bodies and plays important roles in digestion, absorption, and transportation of nutrients in the body, as well as for elimination of waste products and thermoregulation [66]. 

The quantity of water consumed may differ from one person to another and there are no fixed recommendations in this regard. Factors like body size, weather, physical activity, and individual differences have an effect on the body's need for fluids [67]. Usually, thirst is the common indicator of this need, but this mechanism does not always work fully and in many times you may need the water before you get the sign of thirst. Therefore, a general sound recommendation is drinking water and other fluids daily in appropriate amount, especially in the Gulf region, which is known for hot weather and high humidity. However, intake of fluids containing added sugar should be avoided as much as possible. 

Drinking water and other fluids is not the only way to get the required intake of fluids; many of the food consumed contains a good amount of water. For instance, about 85% to 95% of most fruit and vegetable weight is water and meat contains 45% to 65% of water while cheese contains 25–35% water [14]. 

### 3.10. Maintain an Appropriate Weight for Your Height

Obesity has become an epidemic in the Arab Gulf countries during the last two decades. Studies indicate that more than half of adults in these countries are overweight or obese. Meanwhile, the rate of overweight and obesity has risen three-fold among children and adolescents during the same period [12]. The more weight a person has the more the chances of developing hypertension, cardiovascular disease, hypercholesterolemia, and certain kinds of cancers, osteoarthritis and other respiratory problems [68]. Appropriate weight for height is the key to a long healthy life. 

Body mass index (BMI) is often used to measure your healthy weight. BMI is calculated as body weight in kilograms divided by height in meters, squared. People with BMI ranging between 18.5 and 24.9 are considered to be at healthy weight, a BMI between 25.0 and 29.9 to be overweight, and a BMI of 30.0 and over to be obese [69]. 

The major health objective of adults therefore should be to maintain a weight that sustains a healthy life and prevent extra weight gain. The way to achieve a healthy body weight is to balance energy intake (food and drinks) with energy expenditure (physical activity). Excess body fat can be reduced by reducing calorie intake and increasing physical activity [70]. As for children and adolescents, priority should be given to halt the overweight rate along with maintaining natural development. Studies indicate that maintaining a healthy weight for children and adolescents helps to minimize the risk of obesity and its complications in adulthood [8, 68]. Consult your doctor or the dietitian for the appropriate weight of your child. 

### 3.11. Make Physical Activity a Part of Your Daily Routine

Studies in the Arab Gulf countries indicate a serious decline in physical activity especially among women and adults and that these communities tend to be sedentary. It was shown that inactivity is one of the main factors contributing to high prevalence of obesity in the region and that children and adolescents have become less active [13, 43]. 

Physical activity and maintenance of optimum weight are two important issues for good health and both can benefit your health in different ways. Children, adolescents, adults, and elderly people can enjoy wellbeing and improve their health status if they start daily physical activity. Light physical activity includes moving the body about while moderate-intense physical activity is any bodily movement that requires the exertion of much more energy like walking for at least 30 minutes. A target of 30 minutes for adults and 60 minutes for children of moderate physical activity most days of the week, preferably daily, is recommended [70]. 

If you have been practicing physical activity for 30 minutes a day, you can further improve your health by increasing the time of practice. You can practice physical activity at any suitable time, no matter the type or speed of activity of your choice. It is possible to divide time into two periods of 15 minutes each, or to three periods of 10 minutes each. Choose an activity of your choice that you feel you would like to continue. You might see people who prefer to engage in activity relevant to their daily routine, like mowing the lawn or using the stairs, while others prefer to engage in specific activity such as playing football, jogging, or walking. Anyway, engaging in any daily physical activity counts [71]. 

Adults (over 19 years) who suffer from one of the following diseases or have special conditions related to these diseases are advised to seek a medical consultation to check their fitness before they engage in any vigorous activity [72]:

- Existence of chronic disease, for example, heart disease, hypertension, diabetes, osteoarthritis, and obesity.
- Existence of risks leading to heart disease such as smoking, and a rise in blood cholesterol.
- Over 40 years old men and 50 years old women.

### 3.12. Do Not Smoke and Reduce the Risk of Exposure to Smoking Environments

Indicators have revealed an increase in the proportion of smokers in the Arab Gulf countries particularly among adolescents [73]. Moreover, smoking *Shisha* (water pipe) is widely practiced by a considerable proportion of the population at different ages and among different classes [45, 74]. The Arab Gulf communities have begun to accept the smoking of *shisha* among women and adolescents believing wrongly that it is not as harmful as smoking cigarettes. This led to an increase in the use of *shisha* by these groups [45]. 

It is well known that smoking is linked to many heart and respiratory system diseases, as well as to lung cancer, therefore not smoking is highly recommended. Many Muslim scholars have issued claims *(Fatwa)* prohibiting smoking [75]. Smoking *Shisha* is actually worse than smoking cigarettes because the *Shisha's* tobacco contains greater quantities of nicotine and evidence shows that many of its toxicants are insoluble in the *Shisha's* bottled water. Unfortunately, people who are exposed to a smoking environment are also exposed to the same health implications as the smokers themselves. Studies have revealed that a passive smoker is not different from a smoker with regard to the risk of smoking-related diseases [76–78]. Valid advice is not only the cessation of smoking, but also to stay away from smoking environments where there are cigarettes and *Shisha *or any other type of smoking.

### 3.13. Avoid Drinking Alcoholic Beverages

Alcoholic beverage consumption varies in Arab Gulf countries. In some countries, which allow importation of alcoholic beverages the percentage of those who drink alcohol is on the rise, especially among teenagers and young people. In other countries, alcoholic beverages are forbidden, hence these countries witness less alcoholism, nevertheless, economic open-market policies and globalization have contributed to the rise in the number of alcohol drinkers in both environments. Alcohol intake is associated with many diseases, mainly the risks of cirrhosis (hepatitis), hypertension, and esophagus cancer and pancreas infection. In addition, alcoholism damages the heart and cerebrum, increases traffic accident rates, and negatively affects attentiveness at work [79–82]. Alcohol intake during pregnancy has an adverse impact on children's development and behavior and can result in deformity and mental retardation [83]. Alcoholic beverages may contribute to family disagreements and problems such as divorce, violence, and children's misbehavior [79]. 

Although some dietary institutions advice moderate intakes of alcohol and point out some of alcohol's healthy pros, it is worth mentioning that the great majority of local Gulf communities are Muslim (more than 98%) and therefore drinking all types of alcohol is prohibited. 

### 3.14. Ensure Safety of Food Eaten

Recently, emerging food-borne illnesses have begun to constitute a serious concern for medical authorities in all the Arab Gulf countries. The symptoms of such illnesses range from mild to serious and include abdominal cramps, diarrhea, nausea, vomiting and dehydration, and other severe systems of dysfunction such as paralysis and meningitis. Microbial food poisoning is one of the most common types of food-borne diseases that affect many people as a result of eating contaminated foods or foods containing poisons. Symptoms include stomach ache, flatulence, and diarrhea which affect the health of susceptible groups, namely, children and elderly people [41]. 

To avoid microbial illnesses, wash your hands before and after cooking or eating especially when dealing with raw meat. Also, clean surfaces that are directly in contact with food, vegetables, and fruit and do not use these surfaces for chopping up, cutting, or slicing raw meat, chicken, and poultry. It is also important to separate fresh foods from cooked or ready-to-serve foods while shopping, preparing, or freezing. Food should be cooked at a safe temperature, particularly fish, poultry, eggs, meat and its products. Furthermore, it is highly recommended that perishable foods are instantly stored in the refrigerator or freezer and that people follow the proper method of thawing out frozen items before they are cooked. Other important advice is to avoid nonpasteurized milk and raw eggs or dishes which contain these ingredients. 

## 4. Conclusion 

The current FBDG are useful guides for the communities in the Arab Gulf countries to promote their healthy eating and lifestyle to reduce the incidence of nutrition-related diseases among them. However, such guidelines need to be revised periodically (every 3 to 5 years) with the change of scientific evidence and research regarding the risk factors for nutrition-related diseases. We hope that these guidelines will stimulate other investigators in other Arab countries to establish their own ones.

## Figures and Tables

**Table 1:** Food groups and suggested daily servings.

Food Group	Servings	Serving sizes
Cereals and their products	6–11	1 slice, 1/4 Arabic flat bread, 30 g cornflakes, 1/2 cup cooked cereals (rice, wheat oat, macaroni), 6 small crackers (use whole meal cereals).
Vegetables	3–5	1 cup raw leafy vegetables or cooked vegetables, 3/4 cup vegetable juice.
Fruit	2–4	1 medium piece of fruit (banana, apple, mango, pear), 1/2 cup fresh, frozen or canned fruit, 3/4 cup fruit juice.
Milk and dairy products	2-3	1 cup of milk, laban or yoghurt, 43 g of cheese, 1 tablespoon cream cheese (use low fat dairy product).
Meat, chicken, fish, eggs, legumes and nuts	2–4	50–80 g of meat, chicken or fish, one egg, 2 tablespoons of peanut butter, 1/2 cup legumes, 1/3 cup nuts, 2 tablespoons of seeds.

Source: Arab Center for Nutrition [56].
